# Green Extraction Techniques for Obtaining Bioactive Compounds from Mandarin Peel (*Citrus unshiu* var. *Kuno*): Phytochemical Analysis and Process Optimization

**DOI:** 10.3390/foods10051043

**Published:** 2021-05-11

**Authors:** Silvija Šafranko, Ina Ćorković, Igor Jerković, Martina Jakovljević, Krunoslav Aladić, Drago Šubarić, Stela Jokić

**Affiliations:** 1 Faculty of Food Technology Osijek, University of Osijek, Franje Kuhača 18, 31000 Osijek, Croatia; silvija.safranko@ptfos.hr (S.Š.); ina.corkovic@ptfos.hr (I.Ć.); mjakovljevic@ptfos.hr (M.J.); kaladic@ptfos.hr (K.A.); dsubaric@ptfos.hr (D.Š.); 2 Faculty of Chemistry and Technology, University of Split, Ruđera Boškovića 35, 21000 Split, Croatia; igor@ktf-split.hr

**Keywords:** mandarin peel, green extraction, bioactive compounds, bioflavonoids, volatile compounds

## Abstract

In this study, an efficient utilization and valorization of mandarin peel (*Citrus unshiu* Marc. var. *Kuno*) was investigated using innovative and green extraction techniques. The first step of this study included the extraction and analysis of the volatile compounds by performing a supercritical CO_2_ (SC-CO_2_) extraction under different operating pressure conditions (100 and 300 bar). The analysis of volatile compounds of the obtained extracts was conducted by gas chromatography-mass spectrometry (GC-MS), and limonene was found to be the dominant volatile component (13.16% at 100 bar; 30.65% at 300 bar). After SC-CO_2_ treatment, the exhausted citrus peel waste enriched with bioactive compounds was subjected to subcritical water extraction (SWE) in a wide temperature range (130–220 °C) using different solvent-solid ratio (10–30 mL/g) in time periods from 5 to 15 min, in order to obtain bioflavonoids. Identification and quantification of present bioflavonoids was conducted by high-performance liquid chromatography with a with a diode array detector (HPLC), and hesperidin (0.16–15.07 mg/g) was determined as the most abundant flavanon in mandarin peel with other polyphenolic compounds that were possible by-products of thermal degradation. At higher temperatures, the presence of 5-hydroxymethylfurfural (5-HMF) and chlorogenic acid were detected. Antiradical activity and total phenolic content in the extracts were determined using spectrophotometric methods, while the process optimization was performed by response surface methodology (RSM).

## 1. Introduction

The main goal of the industry is to develop and ensure high-quality products followed by the efficient utilization of raw material, minimizing food loss and management costs, reducing also industry waste. The major concern of food-processing industry is efficient waste management and adequate waste disposal [1]. Citrus fruits, which belong to the Rutaceae family, play an important role in food-processing industry and agro-industrial sector, and they were considered as one of the most commonly cultivated and consumed fruits all over the world [2,3]. The main industrial product of citrus processing is undoubtely juice, including also other products such as jams, marmelades, flavouring agents, candied peel and essential oils [4,5,6].

In general, the industrial processing of citrus fruits generates large amounts of organic waste, mainly composed of the peel, pulp, membrane and seed residue [3,7]. Because of the negative environmental impact, it is essential to develop the novel strategies toward the reduction and adequate waste disposal [8,9,10]. Therefore, the novel scientific studies are focused on the valorization of citrus residue with the primary objective of developing of innovative products enriched with bioactive compounds with the emphasis on the efficient waste utilization [5,11,12,13]. Citrus peels contain high contents of vitamin C, fibers, pectins, essential oils and polyphenols (phenolic acids and flavonoids), having strong potential to be exploited as a value-added products, especially for the purposes of food industry, biotechnology and pharmaceutical industry [13,14,15]. Essential oils (EOs) are considered as plant-based natural products comprised of volatile and aromatic compounds widely used in pharmaceuticals, while important commercial application of EOs has been achieved as natural preservatives in food industry [16]. The aromatic profile of citrus peel consists of monoterpenes (e.g., limonene) in majority, following by sesquiterpene hydrocarbons, and their oxygenated derivatives (ketones, aldehydes, alcohols and esters). Limonene is predominant monoterpene in citrus peel, while in lower concentrations linalool, octanal, citronellal can be detected in natural citrus peel essential oils [5,17]. It is well-known that citrus peels are rich in phenolic components, among which flavonoids are most commonly studied due to their beneficial effects to human health [14,15,17]. The most abundant bioflavonoids present in citrus fruits are flavanones, with the predominance of hesperidin with beneficial biological activity exhibiting antioxidant [18], anti-inflammatory [18,19], neuroprotective [20], and antitumor effects [21]. Other important and beneficial flavonoids are narirutin, rutin, diosmin, didymin, sinensetin, and tangeretin which could be detected in small quantites or are available in traces in citrus extracts [22].

In order to obtain the extracts rich in oils, fatty acids or in flavonoids, it is essential to select the appropriate extraction method, primarily solvent and optimize process regarding operating conditions to extract target compounds. Green and sustainable extraction techniques are environmentally-friendly, safe, non-toxic and promising alternatives to the conventional extraction methods. Supercritical CO_2_ extraction (SC-CO_2_) method enables the extraction of volatile and nonpolar compounds due to the nonpolar characteristics of CO_2_ molecules. However, the water in subcritical state (SWE) could obtain extracts rich in flavonoids and phenolic compounds in general, due to the physicochemical changes of solvent under subcritical conditions [5,22]. It is noteworthy to emphasize that there are only few studies reporting SWE methods from citrus peel [5,22,23,24,25], however combination of SC-CO_2_ and SWE extraction with aromatic profile and phenolic compounds characterization and identification from mandarin peel has not yet been reported.

This study is divided into two phases: (a) the first part of experiments was related to SC-CO_2_ extraction of mandarin peel (*Citrus unshiu* Marc. var. *Kuno*) and analysis of volatile compounds by gas chromatography-mass spectrometry (GC-MS); (b) in the second part of experiment the exhausted material remained after SC-CO_2_ method was further used for obtaining phenolic compounds by SWE. The primary hypothesis was that different applied pressures during SC-CO_2_ extraction could obtain aromatic and volatile components of different chemical complexities and the most significant differences in chemical composition of extracts were noticeable at wider ranges of the operating pressures. By removing nonpolar components by SC-CO_2_ extraction, the exhausted material enriched with polyphenolic compounds remained and was extracted by SWE. Qualitative and quantitative analysis of flavonoids was carried out by a high-performance liquid chromatography with a diode array detector (HPLC). The influence of different operating parameters which have the most significant influence during SWE process (temperature, time, and solvent-solid ratio) was investigated, while optimal conditions toward maximizing content of individual flavonoids (desirable components) in the extracts and at the same time minimizing the content of 5-hydroxymethylfurfural (5-HMF) (undesirable component) were also determined. Antiradical activity and total phenolic content in the extracts were determined using standard spectrophotometric methods. This study could be an excellent example of waste valorization by combining two innovative extraction techniques from mandarin peel with the purpose to obtain extracts rich in aromatic as well as extracts rich in phenolic components, respectively.

## 2. Materials and Methods

### 2.1. Chemicals and Plant Material

The mandarin peels (*Citrus unshiu* Marc.) of the variety “Kuno” were obtained in November 2019 from a small family farm Dalibor Ujević (Opuzen, Croatia). Before the extraction, the mandarin peels were washed several times in distilled water, freeze-dried (Alpha LSCplus, Christ, Germany) and milled using a IKA M 20 universal laboratory mill (IKA-Werke GmbH, Staufen, Germany). Carbon dioxide (CO_2_) used in SC-CO_2_ extraction was 99.97% (*w*/*w*) pure (Messer, Osijek, Croatia). The HPLC standard hesperidin (purity 89.5%) was obtained from Dr. Ehrenstorfer GmBH (Augsburg, Germany), while narirutin (purity ≥ 98%), naringin (purity ≥ 95%), and chlorogenic acid (purity ≥ 98%) were purchased from Sigma-Aldrich (Steiheim, Germany). Standards 5-HMF (purity 98%) and rutin (purity 97%) were purchased from Acros Organics (Geel, Belgium). Chemicals 2,2-diphenyl-1-picrylhydrazyl (DPPH) and Folin-Ciocalteu’s phenol reagent were purchased from Sigma-Aldrich Chemie (Steiheim, Germany). All other solvents were of analytical grade (J.T. Baker, Phillipsburg, NJ, USA). Milli-Q pure water was used during experiments and analytical measurements obtained by Milli-Q Millipore system (conductivity ≤ 0.054 μS/cm).

### 2.2. Supercritical CO_2_ (SC-CO_2_) Extraction

In order to obtain the extracts rich in aromatic and volatile compounds, supercritical CO_2_ (SC-CO_2_) extractions were performed in SFE system previously described in details [5,26]. For each experiment, overall 100.0 g of freeze-dried mandarin peel was used, milled in a laboratory mill, and then placed into the extractor vessel made of a stainless steel bar (AISI 304), with the outer diameter (OD) of 100 mm and a height of 500 mm. The extraction experiments were carried out under different pressure conditions (100 and 300 bar), at constant temperature of 40 °C and CO_2_ mass flow rate of 2 kg/h controlled through Matheson FM-1050 (E800) flowmeter (Matheson Tri-Gas, Inc., Basking Ridge, NJ, USA). The extractions were carried out for 90 min, and total extract was collected directly in the glass tube, previously weighted to establish the exact amount of extracts. The obtained extracts were subjected to further GC-MS analysis., and the exhausted material after SC-CO_2_ extraction were kept at 4–6 °C until performing SWE experiments.

### 2.3. GC-MS Analysis

Gas chromatography and mass spectrometry (GC–MS) analyses were performed on a gas chromatograph model 7890A (Agilent Technologies, Palo Alto, CA, USA) with 5975C mass detector (Agilent Technologies, Palo Alto, CA, USA). The operating conditions were: capillary column HP-5MS (5%-phenyl-methyl polysiloxane, 30 m × 0.25 mm i.d., coating thickness 0.25 μm); carrier gas Helium: 1 mL/min; injector temperature: 250 °C; HP-5MS column was heated at 70 °C isothermal for 2 min, and then the temperature was increased to 200 °C at a rate of 3 °C/min and then held isothermal for 18 min; the split ratio: 1:50; ionization voltage: 70 eV; ion source temperature: 230 °C; mass scan range: 45–450 mass units [27]. The extracts obtained by SC-CO_2_ (10 mg) were diluted with hexane and 1 μL of the solution was inserted into the GC injector. The compounds identification was performed by the comparison of their retention indices (RI), determined relative to the retention times of *n*-alkanes (C_9_–C_25_), with those reported in the literature (National Institute of Standards and Technology) and their mass spectra with the Wiley 9 (Wiley, New York, NY, USA) and NIST 17 (D-Gaithersburg) mass spectral libraries. The compounds percentage composition was calculated from the GC peak areas using the normalization method without correction factors. The component percentages were calculated as mean values from duplicate GC-MS analyses of all extracts.

### 2.4. Subcritical Water Extraction (SWE) Technique

The exhausted material remained after SC-CO_2_ procedure at operating pressure of 300 bar was further used as raw material for the extraction of phenolic compounds using subcritical water extraction (SWE) technique after previously removing aromatic and nonpolar components by SC-CO_2_ procedure. SWE extraction experiments were carried out in a handmade SWE system previously described by Jokić et al. [28]. The extraction vessel made from stainless steel bar (AISI 304) was filled with 1.0 g of pretreated plant material of mandarin peel, and nitrogen was injected to prevent oxidation processes during thermal exposure and by the presence of oxygen from air. The experiments were performed by varying operating conditions which are investigated to have possible influence on SWE process: temperature (130–220 °C), time (5–15 min), and solvent-solid ratio (10–30 mL/g). The final product was filtered containing water-soluble phase (extract) and solid residue. The obtained aqueous extracts were further analyzed for phenolic compounds using HPLC technique.

### 2.5. HPLC Analysis

Determination of 5-HMF, bioflavonoids and chlorogenic acid was performed by HPLC (high performance liquid chromatography) method with UV detection, on Cosmosil 5C18-MS-II column (Nacalai Tesque, Inc., Kyoto, Japan), 250 mm long with an internal diameter of 4.6 mm, filled with 5 μm particles. The analysis was performed on a semi-preparative HPLC device (Agilent, 1260 Infinity II series). The HPLC system used for the analysis consisted of a quaternary pump (G7111A), a column chamber (G7116A), a photo-diode array detector (G7115A), an autosampler (G7157A), and a fraction collector (G1364E). The system was operated using a computer program Prep LC Online. The separation of analyzed compounds was performed by gradient elution for 50 min, where 1% CH_3_COOH (in Milli-Q water) was used as the phase A and methanol as the phase B, with 80:20 ratio of A:B. The flow rate was 1.0 mL min^−1^, the injection volume was 20 µL, the UV detection wavelengths were 283 and 360 nm, and the analysis was performed at 25 °C. The gradient conditions were: 0–5 min 80% of A, 5–15 min 80–40% of A, 15–35 min holding 20% of A, 35–40 min 20–40% of A, 40–50 min 40–80% of A. The standard stock solution for hesperidin was prepared in dimethyl sulfoxide (DMSO) and diluted with methanol, and for other components the standard solutions were prepared in methanol. The compounds 5-HMF, hesperidin, narirutin and chlorogenic acid were detected at 283 nm, while rutin was detected at 360 nm. The calibration for 5-HMF was obtained at seven concentrations (10–1000 mg/L), for hesperidin at seven concentrations (1.0–1500 mg/L), for narirutin at six concentrations (10–150 mg/L), for rutin at seven concentrations (10–500 mg/L) and for chlorogenic acid at eight concentrations (10–1000 mg/L). The linearity of the calibration curve was confirmed by R^2^ = 0.99927 for 5-HMF, R^2^ = 0.99920 for hesperidin, R^2^ = 0.99888 for narirutin, R^2^ = 0.99842 for rutin and R^2^ = 0.99912 for chlorogenic acid. The retention time for 5-HMF was 5.221 min, for chlorogenic acid 12.333 min, for narirutin 18.368 nm, for hesperidin 18.838 min and for rutin 19.034 min.

The sample analysis was performed in duplicate, and two injections were performed from each prepared solution. The compounds content in the analyzed samples were expressed in mg/g of peels (further in the text expressed as mg/g).

### 2.6. Determination of Antiradical Activity

Determination of antiradical activity of SWE extracts of mandarin peel was performed by standard DPPH spectrophotometric method [29]. A volume of 0.5 mL of freshly prepared DPPH methanolic solution (0.3 mM) was added to the 1.2 mL of the extract (1.0 mg/mL). The reaction mixtures were kept in dark for 30 min before the measurements. The absorbances were measured at 517 nm (Spectronic Helios Gamma UV-Vis spectrophotometer; Thermo Fisher Scientific, Waltham, MA, USA), and all experiments were performed in three replicates, while the results are expressed as mean percentage inhibition ± standard deviations.

### 2.7. Determination of Total Phenolic Content

The content of total phenols of SWE extracts of mandarin peel was determined by spectrophotometric method using Folin-Ciocalteu reagent. Briefly, in 200 μL of SWE extracts (10.0 mg/mL) was added 100 μL of Folin-Ciocalteu reagent, 300 μL of 20% Na_2_CO_3_ aqueous solution, and then Milli-Q water (conductivity ≤ 0.055 μS/cm) was added to the mixture to the overall volume of 2 mL of the mixture. Methanol was used as a blank sample. Finally, the reaction mixtures were incubated at 40 °C for 30 min. For the preparation of calibration curve, gallic acid was used as standard compound. The absorbances were measured at 765 nm using a Shimadzu UV-1280 spectrophotometer (Shimadzu, Kyoto, Japan). The obtained results were expressed as mg of gallic acid equivalent per g of peel material (mg GAE/g). All experiments were carried out in triplicate and the results are expressed as mean values ± standard deviations.

### 2.8. Experimental Design and Process Optimization

Experimental data generated by Box-Behnken design (BBD) [30] and response surface methodology (RSM) were used to derive optimal process parameters and to evaluate model adequacy for each individual response (*y*) obtained by SWE extraction of the citrus waste material pretreated by SC-CO_2_ procedure. The extraction parameters such as temperature (*X*_1_), time extraction (*X*_2_) and solvent-solid ratio (*X*_3_) are independent variables investigated for the influence of individual variable on the SWE process (Table 1), more precisely on the dependent variable or response (*y*). Obtained data were fitted with a second order (quadratic) response models described by the following Equation (1):

(1) $$y=\beta_{0}+\sum_{i=1}^{k}\beta_{i}X_{i}+\sum_{i=1}^{k}\beta_{ii}X_{i}{}^{2}+\sum_{\begin{array}{l} i=1\\ i<j \end{array}}^{k-1}\sum_{j=2}^{k}\beta_{ij}X_{i}X_{j}$$

where *y* represents set of investigated responses (target bioactive compounds in SWE extracts), *β*_0_, *β_i_*, *β_ii_*, *β_ij_* parameters are designated as constant coefficients of intercept, linear, quadratic, and interaction terms, respectively; *X_i_* and *X_j_* are coded (−1, 0, +1) inputs or independent variables. The response of chlorogenic acid was fitted to the reduced cubic model without transformation. The process optimization was performed for the three identified and most abundant phenolic components (hesperidin, narirutin, and rutin) and possible degradation products chlorogenic acid and 5-HMF detected in SWE extracts, while evaluation of the developed models was done by performing experiments according to the calculated optimal operating conditions. The statistical analysis was carried out using commercial software Design-Expert^®^ (ver. 9, Stat-Ease Inc., Minneapolis, MN, USA). The quality of fitted mathematical models was evaluated by the analysis of variance (ANOVA).

## 3. Results and Discussion

By combining two innovative extraction techniques, SC-CO_2_ and SWE, total reusage of the citrus waste material could be achieved in order to obtain the valuable compounds. By applying different operating pressures in SC-CO_2_ extraction procedure, nonpolar compounds of different complexities could be obtained [5]. The increase in the extraction pressure causes the increase in the SC-CO_2_ density and solvent solubility, enhancing its extracting power and process yield [31]. Hence, in order to compare the efficiency of the extraction on the yield and the composition of volatile components by SC-CO_2_ method, the variations in two operating pressures (100 and 300 bar) were employed. It is well-known that volatile and low-molecular-weight compounds could be extracted under lower applied pressure (essential oils), while with the increase of pressure in the system less volatile and higher molecular weight could be obtained (fatty acids, waxes, etc.) [32]. Two pressures in wide range (100, 300 bar) were used in the SC-CO_2_ extraction process, while temperature and CO_2_ flow rate were kept constant due to the many years of author’s experience in the field of SC-CO_2_ extraction and the fact that temperature and pressure have no significant effect on the final product. The applied temperature of 40 °C is a temperature where CO_2_ is in supercritical state, but the temperature is low enough to prevent degradation of thermolabile compounds, preventing also damage of their structure. By removing aromatic and volatile compounds from the citrus material, the extractability of the remained bioflavonoids could be enhanced by using SWE. It has been also reported that at the temperature of 150 °C, dielectric constant (ε) of water decreases and chemical characteristics of solvent is equal to those of DMSO at 25 °C, which means that compounds of different polarity could be obtained by varying operating conditions of SWE [22]. The first observations in this study were made according to the physical appearance of the extracts obtained by SWE. The color of the obtained extracts changed from characteristic orange color to brown color depending on the applied temperature. This color derived from both components found in the citrus peel as well as from by-products of Maillard reactions. The color appeared more intense and dark brown with the increase in the extraction temperature, while Cvetanović et al. [33] reported that the rate of this reaction increases significantly between 140 °C and 160 °C.

### 3.1. GC-MS Analysis of SC-CO_2_ Extracts

The most abundant aromatic compound, as expected was monoterpene hydrocarbon limonene (13.16%; 30.65%), being more abundant at 300 bar. It was previously noted that the percentages of limonene, as the major typical volatile compound of *Citrus* peels, in general was lower than in pressed *Citrus* peel oils [27], but SC-CO_2_ extraction provided enriched profiles of volatiles and semi-volatiles with respect to oxygenated monoterepenes and sesquiterpenes. Lopresto et al. [34] performed the extractions on the lemon peel in order to obtain the extracts rich in volatile compounds. They reported that hydrodistillation gave the isolate enriched in monoterpene limonene, with superior performance compared to SC-CO_2_ extraction. The authors emphasized that the possible reason of the lower recovery of limonene (17.97%; 35.71%) obtained by SC-CO_2_ extraction is due to the lower selectivity of SC-CO_2_ toward monoterpenes. With the increase of operating pressure from 100 to 300 bar, the solvent power has enhanced, extraction selectivity is lower, and significant content of waxes could be extracted causing the decrease in content of essential oils in SC-CO_2_ extract. Another more abundant monoterpenes (Table 2) were: γ-terpinene, linalool and α-terpineol. On the other hand, among sesquiterpene hydrocarbons, the most abundant were α-farnesene (10.63%; 5.72%), germacrene D (6.66%; 4.11%), eremophilene (6.7%; 3.99%), followed by γ-cadinene, germacrene B and others (Table 2). Another group of highly abundant compounds were fatty acids, particularly linoleic acid and hexadecanoic acid (Table 2). However, those acids are not typical flavor compounds of the citrus peels. The obtained results are partially similar to the SC-CO_2_ extraction at 40 °C and 10 MPa at 1.76 kg/h of the peels of *Citrus aurantium* L. and *Citrus sinensis* Osbeck cultivars from the Dubrovnik region (south Croatia) with relevant similarities among the peel oil compositions of *C. aurantium* and *C. sinensis* cultivars with limonene predominance (up to 54.3%) [33]. The principal present oxygenated monoterpenes were linalool (3.0–5.9%), α-terpineol (0.7–2.4%), linalyl acetate (0.0–5.0%), geranyl acetate (0.0–0.4%), (*Z*)-citral (0.0–1.8%) and (*E*)-citral (0.0–1.9%), while several sesquiterpenes were found with minor percentages. However, coumarin derivatives (isogeijerin, scoparone, bergapten, or osthole, 7-methoxy-8-(2-formylpropyl)coumarin) found among relevant compounds in mentioned research were not identified in the present research.

### 3.2. Identification and Quantification of the Compounds in SWE Extracts Using HPLC

The mandarin peel residue after SC-CO_2_ extraction was used for further extraction of more polar components with subcritical water and their content was determined by HPLC. The residue obtained by SC-CO_2_ extraction performed at 300 bar was selected for further investigation of polyphenolic compounds as higher recovery of limonene and content of fatty acids was observed. Overall of 17 extractions were performed according to the BBD experimental design using SWE. The results have indicated that the significant concentrations of the compounds 5-HMF, hesperidin, narirutin, rutin, and chlorogenic acid were detected in the obtained extracts. The results of individual components are expressed as mg/g of the peels (exhausted material) after CO_2_ extraction and are shown in Table 3.

It is well known fact that the composition of the extract, as well as the amount of individual components, depended also on the extraction conditions as can be seen in Table 3. Given the noticeable differences in the number and quantity of components, the chromatographic profile of extracts, obtained under the following conditions of 220 °C, time of 10 min, using 30 mL/g ratio (sample 17), and the extract obtained at 130 °C, 15 min, and 20 mL/g are selected and presented in Figure S1 (sample 8). As can be seen from Figure S1, five major and several minor peaks were observed at 283 nm, of which five were identified. The largest peak was identified as 5-HMF (Figure S1b, peak 1). The second peak was identified as chlorogenic acid, the third peak was hesperidin, while the fourth was naringenin. Peak identification was performed by comparing retention times and UV spectra with commercial standards (Figure S2). In addition to the appearance of the UV spectrum, the correspondence between the spectra of the standards of unknown peaks is shown, with a coincidence of 82.21% to 98.00% depending on the component (Figure S2). In this way, comparing the retention times and UV spectra the identity of the components was confirmed, while the concentrations of detected components were calculated by reference to the external standard curve constructed using various concentrations of each of the commercially available standard compounds.

As presented in Table 3, 5-HMF was detected in almost all samples except in those obtained at lower temperatures (130 °C). The amount of 5-HMF ranged from 0.00 mg/g to 14.33 mg/g. The highest concentration was obtained in the sample 17 (temperature 220 °C, time 10 min, solvent-solid ratio 30 mL/g), and the lowest in the samples 8 (temperature 130 °C, time 15 min, solvent-solid ratio 20 mL/g) and 9 (temperature 130 °C, time 10 min, solvent-solid ratio 10 mL/g). Since it is a compound formed through Maillard and/or caramelization reactions, it can be found in most of the thermal processing of food, especially in carbohydrate-rich foods [35]. In the previous studies of the extractions of citrus, especially mandarin peel [22,36], there is no data presented on 5-HMF in SWE samples to compare the results, but the extracts of the lemon peel showed an increase in the amount of 5-HMF with increasing temperature and extraction time. The highest amount of 5-HMF (231.21 ± 3.14 mg/L) was found in lemon peel extract at 180 °C and 45 min. 5-HMF in lemon peel extract was determined at 0.64 mg/L at 140 °C during SWE but its content in lemon peel extracts increased 20.8 times with the rise of temperature from 140 °C to 160 °C for 15 min during SWE. The temperature of 180 °C has been shown to be the critical temperature for 5-HMF formation in lemon peel extraction, which can be explained by the decomposition of hemicellulose comprised of different sugar units, among which lemon peel consists of 8.09% dry weight. Also, the increase in 5-HMF with increasing temperature can be explained by the decomposition of the cell wall at high temperatures and with high lignin content (7.56%) of lemon peel [37].

Hesperidin and narirutin were the most abundant flavanones present in the extracts. The concentration of hesperidin detected in the extracts ranged from 0.16 mg/g to 15.07 mg/g. Furthermore, the lowest concentration was observed in the sample 14 (temperature 220 °C, time 15 min, solvent-solid ratio 20 mL/g), and the highest in the sample 10 (temperature 175 °C, time 5 min, solvent-solid ratio 30 mL/g). The concentration of hesperidin is significantly higher in the extracts obtained at 130 °C and 175 °C than at applied higher limit temperature of 220 °C. The concentration of narirutin in the extracts ranged from 0.03 mg/g to 5.11 mg/g. The lowest concentration was observed in sample 7 (temperature 220 °C, time 10 min, solvent-solid ratio 10 mL/g), and the highest concentration in sample 16 (temperature 175 °C, time 10 min, solvent-solid ratio 20 mL/g). Comparing the results with the literature, it can be seen how other authors have done similar extractions and analyzes. Cheigh et al. [36] recorded the highest yield of hesperidin (73.5 ± 0.5 mg/g of the plant) and narirutin (11.7 ± 0.8 mg/g of plant) at the extraction temperature of 160 °C and solid-liquid ratio of 34 mL/g of mandarin peel. Bioactive flavonoids from *Citrus unshiu* peel were extracted using subcritical water in a semi-continuous mode. With this method of extraction, the extraction temperature and flow rate are optimized as the most important parameters. Therefore, the highest yield of hesperidin (44.62 mg/g of dry sample) was achieved at 160 °C and 2.25 mL/min during 15 min, and narirutin (8.00 mg/g) at 150 °C and 2 mL/min during 15 min. Under extraction conditions of 175 °C, for 15 min and a flow rate of 1.5 mL/min, the yields of hesperidin and narirutin of 34.75 mg/g from the dry sample and 6.60 mg/g were achieved respectively [38]. In another study, the efficiency of hesperidin and narirutin extraction from *Citrus unshiu* peel using SWE with the extraction temperatures from 110 °C to 190 °C and the extraction times from 3 min to 15 min were examined. The best yield of hesperidin (38.45 mg/g dry peel) was achieved at 150 °C and 15 min, and for narirutin (6.56 mg/g dry peel) it was achieved at 190 °C and 5 min. The extraction efficiency was improved by a combination of a pulsed electric field (PEF) and SWE where the concentration of hesperidin was 46.96 ± 3.37 mg/g peel and narirutin 8.76 ± 0.83 mg/g peel [24].

The amount of rutin detected in the extracts was in the range of 0.18 mg/g to 4.27 mg/g. The lowest concentration of rutin was detected in sample 14 prepared under extraction conditions of 220 °C, time of 15 min, and with solvent-solid ratio of 20 mL/g. The highest concentration was detected in sample 16 prepared under extraction conditions of 175 °C, 10 min, and at solvent-solid ratio of 20 mL/g.

In addition, chlorogenic acid as a dominant phenolic acid in the phytochemical profile of SWE extract of *Citrus unshiu* Marc. var. *Kuno* was also detected in several samples. The detected values of chlorogenic acid concentration in the prepared extracts ranged from 0.00 mg/g to 68.58 mg/g. The lowest concentration of chlorogenic acid was recorded in sample 8 (temperature 130 °C, time of 15 min, solvent-solid ratio 20 mL/g), and the highest for sample 17 (temperature 220 °C, time of 10 min, solvent-solid ratio 30 mL/g). This increase in chlorogenic acid can be explained by the degradation of lignin during subcritical water extraction to form secondary metabolites such as flavonoids (luteolin and apigenin) and phenolic acids (chlorogenic acid and dicaffeoylquinic acids). These components are precursors during lignin biosynthesis [39].

### 3.3. Response Surface Analysis and Process Optimization

**Figure 1:**
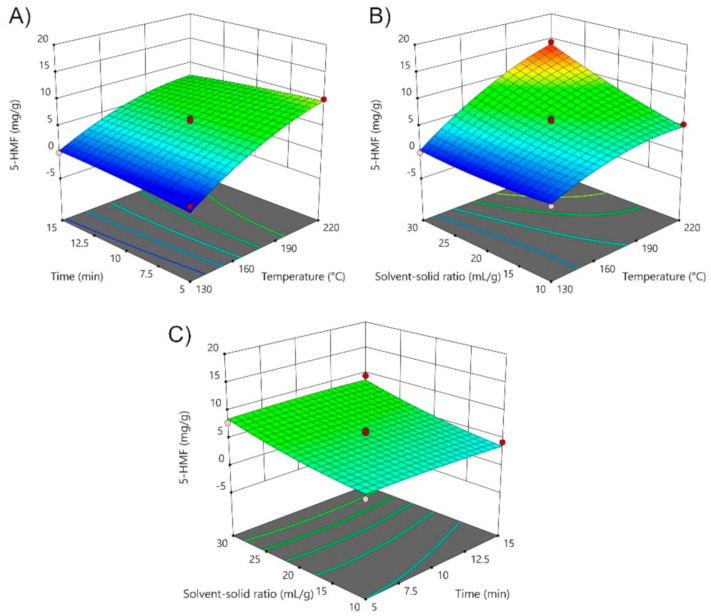
3D diagrams of the effects of (**A**) time and temperature; (**B**) solvent-solid ratio and temperature, and (**C**) solvent-solid ratio and time on the 5-HMF extraction from mandarin peel (*Citrus unshiu* Marc. var. *Kuno*) using SWE technique.

In order to optimize the extraction process for obtaining the most abundant bioflavonoids presented in mandarin peels (*Citrus unshiu* Marc. var. *Kuno*) by employing SWE technique, it is essential to investigate the effects of the operating parameters (temperature, time and solvent-solid ratio) on the individual response (targeted compound). In this study, SWE has been used for the extraction of polyphenolic compounds from the SC-CO_2_ pretreated residue of mandarin peel, while the influence of the operating parameters has been investigated regarding to the three the most abundant polyphenolic components in the samples, more precisely, to hesperidin, narirutin, and rutin, as well as to chlorogenic acid and heterocyclic organic compound 5-HMF. The mathematical modeling and process optimization were performed according to the Box-Behnken experimental design, testing also the influence of three process parameters of temperature (130–220 °C; *X*_1_), time (5–15 min; *X*_2_) and solvent-solid ratio (10–30 mL/g; *X*_3_) through overall 17 experimental runs (Table 1 and Table 3). As expected, the concentration of 5-HMF increases with the increase of extraction temperature, with a maximum concentration detected at 220 °C (14.33 mg/g of peels). By investigating the influence of each individual process parameter on 5-HMF (Figure 1), temperature (*X*_1_) and solvent-solid ratio (*X*_3_) show significant effect on the presence of 5-HMF in the extracts (Table S1). All temperature coefficients and their interactions (*X*_1×3_, *X*_1_^2^) exhibit also significant influence on the presence of 5-HMF, and as it can be seen, the regression model for the response of 5-HMF was significant (*p*-value < 0.05) and obtained coefficient of determination was calculated to be R^2^ = 0.9677. The Equation (2) for the following model was determined as:

(2) $$5-HMF=5.95+4.52X_{1}-0.1786X_{2}+2.28X_{3}-1.61X_{1}^{2}-0.2184X_{2}^{2}\;\;\;\;\;\;\;\;+\;0.5871X_{3}^{2}-0.9153X_{1}X_{2}+2.23X_{1}X_{3}+0.25X_{2}X_{3}$$

The concentration of 5-HMF at 130 °C is negligible, reaching its maximum at the highest applied temperature of 220 °C. 5-HMF is in the most cases considered as one of the by-products obtained by thermal degradation of present components in the extracts or by non-enzymatic browning. According to the Lachos-Perez et al. [22], during treatments at high temperatures, the compounds present in plant material could mutually interact to give different by-products, among which 5-HMF is the most commonly detected. However, due to its toxic effect at high concentrations, it is essential to optimize the process in order to reduce its concentration in the extracts, while at the same time to increase the concentration of desirable compounds, such as polyphenolic components. Also, the level of 5-HMF is monitored as an index of nonenzymatic browning and could be used as indicator of quality loss in food products [40].

**Figure 2:**
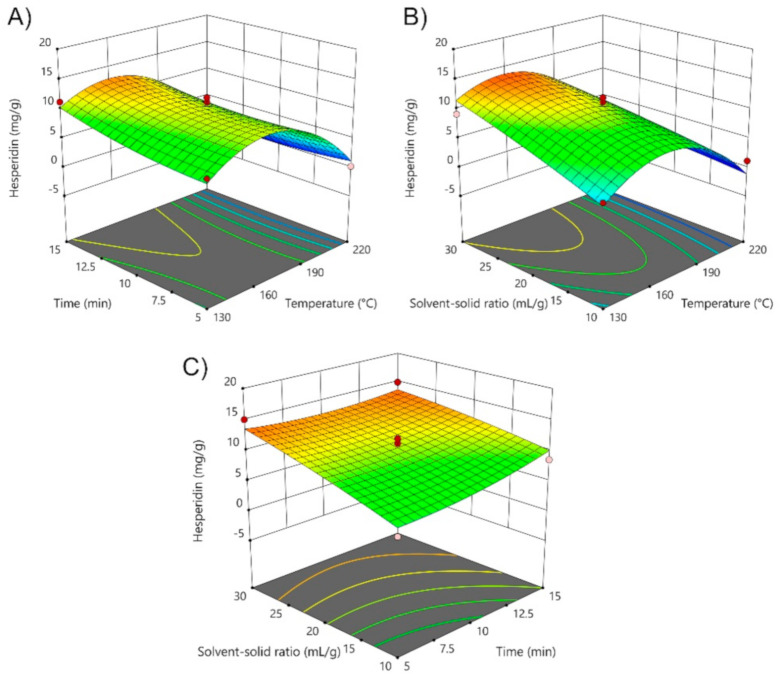
3D diagrams of the effects of (**A**) time and temperature; (**B**) solvent-solid ratio and temperature, and (**C**) solvent-solid ratio and time on hesperidin extraction from mandarin peel (*Citrus unshiu* Marc. var. *Kuno*) using SWE technique.

Hence, the influence of operating extraction parameters was also investigated on the extractability of the three most abundant components in SWE extracts (Figure 2, Figures S3 and S4), hesperidin, narirutin and rutin.

**Figure 3:**
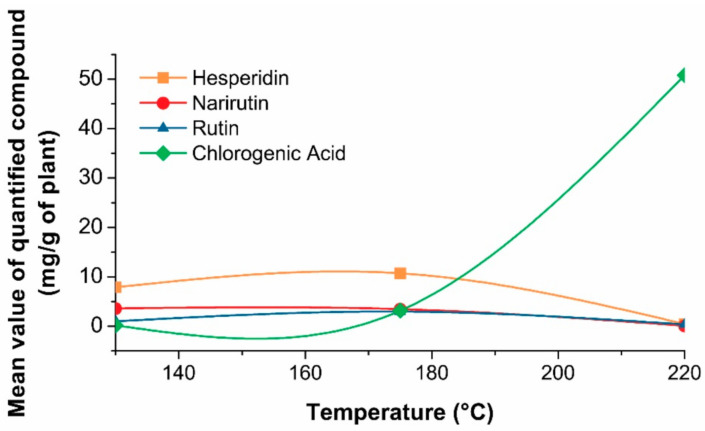
The effect of temperature on the recovery of the three most abundant phenolic compounds and chlorogenic acid in SWE extracts.

In the case of the hesperidin and narirutin, the parameters such as temperature (*X*_1_) and solvent-solid ratio (*X*_3_) have significant influence on the extraction of each individual bioflavonoid as it can be noticed from the results of ANOVA analysis summarized in Tables S2 and S3. The temperature (*X*_1_) parameter did not show significant effect, while quadratic coefficient of temperature (*X*_1_^2^) and solvent-solid ratio (*X*_3_) show significant influence on the extraction of rutin (Table S4). The common characteristic for all investigated responses is the significant influence of the solvent-solid ratio (*X*_3_), meaning that as more solvent enters the plant cells, the extraction of phenolic compounds by permeating the cell membrane will be enhanced under higher solvent-solid ratios [41]. Another common characteristic is relatively low stability of flavonoids at the higher temperatures than >175 °C, indicating the decrease in concentrations by possible thermal degradation of components. Therefore, the maximum contents of individual response are obtained under the highest solvent-solid ratio of approximately 30 mL/g and temperatures ≤ 175 °C. An interesting observation has been made regarding the increasing concentration of chlorogenic acid with the increase of temperature (Figure S5). The highest concentration of chlorogenic acid of 68.58 mg/g of peels has been detected in the experimental run 17 (220 °C, 10 min, 30 mL/g of peels) of SWE extract of mandarin peel, while significant decrease in concentrations of flavonoids hesperidin, narirutin and rutin has been observed (Figure 3). These findings suggest that chlorogenic acid could be formed as a by-product of thermal degradation of the present components in the extracts at higher temperatures.

The adequacy of the fitted models has been evaluated by the ANOVA analysis shown in Tables S1–S5 (reported in Supplementary Material). As it can be noticed, the models for the extraction of hesperidin, narirutin and rutin are significant (*p*-value < 0.05) with obtained coefficient of determination ranging from R^2^ = 0.8487 to R^2^ = 0.9317, showing good correlation between fitted and experimental data. The obtained results fit to the quadratic function described by following equation for hesperidin (3), narirutin (4), rutin (5), while chlorogenic acid (6) is fitted to the cubic function as follows:

(3) $$HESPERIDIN=10.53-3.7X_{1}+0.873X_{2}+2.61X_{3}-6.58X_{1}^{2}+0.7849X_{2}^{2}\;\;\;\;\;\;\;\;-\;0.3627X_{3}^{2}-0.9838X_{1}X_{2}-1.58X_{1}X_{3}-0.8629X_{2}X_{3}$$

(4) $$NARIRUTIN=3.84-1.77X_{1}-0.0268X_{2}+0.9936X_{3}-1.57X_{1}^{2}-0.0753X_{2}^{2}\;\;\;\;\;\;\;\;-\;0.7832X_{3}^{2}-0.2785X_{1}X_{2}-0.4381X_{1}X_{3}+0.1045X_{2}X_{3}$$

(5) $$RUTIN=3.24-0.3091X_{1}-0.1395X_{2}+0.7244X_{3}-2.29X_{1}^{2}-0.3365X_{2}^{2}\;\;\;\;\;\;\;-\;0.2277X_{3}^{2}-0.002X_{1}X_{2}-0.3326X_{1}X_{3}+0.123X_{2}X_{3}$$

(6) $$\begin{array}{c} CHLOROGENIC\;ACID\\ \;\;\;\;\;\;\;\;\;=3.16+22.37X_{1}+11.78X_{1}X_{3}+22.33X_{1}^{2}+11.88X_{1}^{2}X_{3}\\ \;\;\;\;\;+\;5.91X_{1}X_{2}^{2}+1.57X_{2}^{2}+3.57X_{2}X_{3}^{2}-1.19X_{2}^{3} \end{array}$$

One of the main focus of this study is to optimize SWE process by maximizing the content of three most abundant flavonoids in mandarin peel, including also chlorogenic acid, and by minimizing content of undesirable compound 5-HMF using desirability function approach. For obtaining the maximum content of hesperidin and minimum content of 5-HMF by SWE procedure within experimental range, the calculated optimal conditions were 130 °C, 14 min, and 29 mL/g. The optimal conditions for the separation of narirutin (4.92 mg/g) were 130 °C, 14 min, and 27 mL/g and 145 °C, 8 min, and 29 mL/g for obtaining rutin (3.10 mg/g). As it can be noticed, the optimal conditions values are close to the lower limits of experimental design (130 °C), and are undoubtedly necessary to apply in order to reduce higher concentrations of 5-HMF in the SWE extracts, as 5-HMF is a product of sugar transformation under high temperature conditions. The predicted data obtained with RSM analysis was experimentally verified with satisfactory agreement to the experimental values within deviations of ±10%. The prediction was also verified excluding the condition of minimizing the content of 5-HMF, and results have shown the optimal conditions for maximum content of hesperidin (15.05 mg/g) as 153 °C, 15 min, and 30 mL/g, 140 °C, 15 min, and 29 mL/g for narirutin (5.05 mg/g), 168 °C, 10 min, and 30 mL/g for rutin (3.79 mg/g) and 219 °C, 9 min, and 30 mL/g for maximum content of chlorogenic acid (68.76 mg/g). The experimental data show good agreement to the predicted data with suitable deviations of ±5%.

### 3.4. Antiradical Activity and Total Phenolic Content

**Figure 4:**
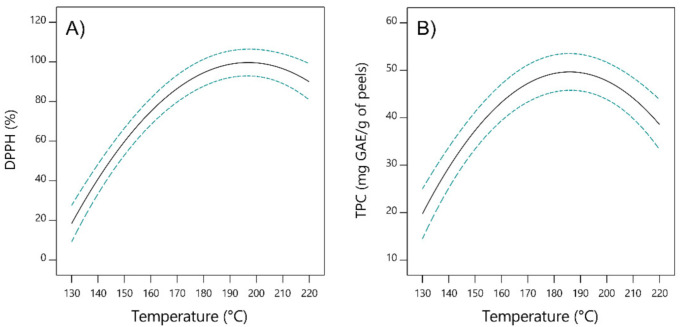
2D diagram of the effect of the applied extraction temperature on (**A**) antiradical activity and (**B**) total phenolic content (TPC).

The extracts obtained by SWE were analyzed for antiradical activity and total phenolic content. It is well-known that the antioxidant capacity is enhanced in hydrophilic environment, especially in the presence of the phenolic compounds, and could be reduced in lipophilic environment, such as in SC-CO_2_ extracts [42]. SWE extracts of mandarin peel exhibited good antiradical activity within the range of 11.14 ± 5.32% and 95.28 ± 0.37%. The highest antiradical activity was detected in the sample obtained at 175 °C, 15 min, and at solvent-solid ratio of 30 mL/g (run 5). The antiradical activity significantly increases with the increase of temperature from 130 °C to 175 °C, while decrease in activity is observed with further increase in temperature above 200–220 °C (Figure 4A).

This clearly indicates that both simple and complex polyphenolic compounds contribute to the antiradical activity, while at the higher temperatures above 160 °C, degradation process of the polyphenolic compounds initiates, mainly of hesperidin and narirutin, which are well known for their good antioxidant capacity [25]. Similar observation was made regarding determination of total phenolic content. At mild conditions of 130 °C, 5 min and 20 mL/g, total phenolic content was determined as 15.80 ± 1.32 mg GAE/g of peels, while the highest content of 54.39 ± 0.63 mg GAE/g of peels was detected at the 175 °C, 10 min, 20 mL/g (Figure 4B). The results could be attributed to the greater solubility of phenolic compounds, while at the same time physical characteristics of the contact surface also change (surface tension, viscosity), improving their solubility in the solvent. The correlation between two variables, antiradical activity and total phenolic content, has been also investigated and correlation coefficient was calculated to be R = 0.8823. These findings indicate that SWE extracts exhibit good antiradical activity with significant content of phenolics, presenting citrus peel extracts as promising plant material for a wide spectrum of applications and potential health benefits.

## 4. Conclusions

This study focuses on the innovative green extraction techniques of the mandarin peel (*Citrus unshiu* Marc. variety *Kuno*) to obtain the highly valuable components. Firstly, SC-CO_2_ extractions at operating pressures of 100 and 300 bar were performed to obtain aromatic components of different chemical complexities, among which limonene was detected as the most dominant volatile compound at both applied pressures, followed by α-farnesene, linoleic and hexadecanoic acids. The exhausted residue of mandarin peel remained after SC-CO_2_ extraction was further used for extraction of polyphenolic compounds using SWE. Based on the obtained results, the most abundant phenolic components in mandarin peel extracts obtained by SWE were hesperidin, narirutin, and rutin. The concentration of these components was dependent on the applied temperature and solvent-solid ratio, with significant decrease observed in extracted content above 160 °C. At these conditions, the higher contents of chlorogenic acid and 5-HMF were detected, which are suggested as by-products of thermal degradation. The extracts obtained at higher temperatures ≤175 °C exhibited good antiradical activity, however the formation of 5-HMF was also observed. Potential limitation of SWE could be attributed to the formation of undesirable components by applying the higher extraction temperatures. Hence, efficient optimization process for obtaining highly valuable and at the same time reducing the content of undesirable components is undoubtedly a study of interest and essential toward possible large-scale applications.

## Figures and Tables

**Table 1:** The uncoded and coded levels of independent variables used in the RSM design for SWE technique from citrus material pretreated by SC-CO_2_ extraction.

Independent Variable	Symbol	Level		
		Low (−1)	Center (0)	High (+1)
Temperature (°C)	*X*_1_	130	175	220
Extraction time (min)	*X*_2_	5	10	15
Solvent-solid ratio (mL/g)	*X*_3_	10	20	30

**Table 2:** The volatiles profile of SC-CO_2_ extracts * of mandarin peel var. *Kuno* determined by GC-MS analysis.

No.	Compound	100 Bar (%)	300 Bar (%)	No.	Compound	100 Bar (%)	300 Bar (%)
1.	α-Pinene	0.03	0.09	27.	Undecanal	0.09	0.06
2.	Sabinene	-	0.04	28.	δ-Elemene	0.31	0.4
3.	Hexanoic acid	0.05	0.03	29.	α-Cubebene	0.11	0.08
4.	β-Myrcene	0.13	0.54	30.	Citronellyl acetate	0.24	0.15
5.	α-Terpinene	-	0.04	31.	Neryl acetate	0.43	0.28
6.	*p*-Cymene	0.04	-	32.	α-Copaene	1.46	0.89
7.	Limonene	13.16	30.65	33.	Geranyl acetate	0.72	0.45
8.	(*Z*)-β-ocymene	0.02	0.05	34.	β-Elemene	0.99	0.86
9.	γ-Terpinene	1.75	3.69	35.	Dodecanal	0.23	0.13
10.	*cis*-Sabinene hydrate	0.11	0.16	36.	*trans*-Caryophyllene	0.9	0.54
11.	α-Terpinolene	0.17	0.32	37.	Valencene	0.51	0.3
12.	Linalool	2.18	1.58	38.	α-Muurolene	0.63	-
13.	Nonanal	0.07	0.06	39.	Eremophilene	6.7	3.99
14.	*trans*-*p*-mentha-2,8-dien-1-ol	-	0.02	40.	γ-Cadinene	2.21	1.38
15.	*trans*-Limonene oxide	0.02	0.02	41.	Germacrene B	1.11	0.69
16.	Citronellal	0.11	0.1	42.	Dodecanoic acid	0.34	0.32
17.	Terpinen-4-ol	0.22	0.16	43.	*t*-Muurulol	0.07	0.06
18.	Octanoic acid	0.08	0.05	44.	3-Oxo-α-ionol	-	-
19.	α-Terpineol	2.1	1.31	45.	α-Sinensal	0.08	0.05
20.	Decanal	0.53	0.37	46.	Tetradecanoic acid	2.42	1.56
21.	β-Citronelol	0.19	0.13	47.	Nootkatone	0.14	0.13
22.	Perilla aldehyde	0.43	0.3	48.	Octadecan-1-ol	0.18	0.17
23.	Nonanoic acid	0.08	0.06	49.	Heptadecanoic acid	0.19	0.4
24.	*p*-Mentha-1,8-dien-9-ol	0.23	0.15	50.	Linoleic acid	15.44	19.04
25.	Thymol	0.09	0.08	51.	Oleic acid	2.87	-
26.	Carvacrol	0.19	0.12				

* extraction temperature: 40 °C.

**Table 3:** Experimental design for SWE procedure and obtained contents of polyphenolic compounds analyzed by HPLC.

Experimental Design (BBD)				Compound (mg/g of Peels)				
Run	Temperature (°C)	Time (min)	Solvent-Solid Ratio (mL/g)	5-HMF	Hesperidin	Narirutin	Rutin	Chlorogenic Acid
1.	175	10	20	4.68	9.28	3.65	3.14	0.27
2.	175	15	10	4.32	8.56	1.05	0.89	1.90
3.	130	10	30	0.01	9.19	3.83	1.31	0.28
4.	220	5	20	10.08	0.19	0.09	0.19	58.93
5.	175	15	30	9.48	14.89	4.27	3.91	8.69
6.	175	10	20	6.48	10.52	3.63	3.03	3.62
7.	220	10	10	5.38	1.15	0.03	0.80	21.26
8.	130	15	20	0.00	11.25	4.87	1.04	0.00
9.	130	10	10	0.00	3.66	1.99	0.52	0.08
10.	175	5	30	7.83	15.07	4.72	4.21	0.28
11.	175	5	10	3.67	5.28	1.91	1.68	0.79
12.	175	10	20	6.18	9.61	3.44	2.94	1.98
13.	175	10	20	6.02	11.23	3.38	2.81	1.84
14.	220	15	20	6.39	0.16	0.08	0.18	54.48
15.	130	5	20	0.03	7.34	3.76	1.04	0.29
16.	175	10	20	6.41	11.99	5.11	4.27	9.10
17.	220	10	30	14.33	0.35	0.11	0.26	68.58

## Data Availability

The data presented in this study are available for limited time on request from the corresponding author.

## Supplementary Materials

The following are available online at https://www.mdpi.com/article/10.3390/foods10051043/s1, Figure S1. HPLC chromatograms; Figure S2. Comparison of UV spectra of standards and unknown peaks; (A) hesperidin, (B) narirutin, (C) rutin, (D) 5-HMF, and (E) chlorogenic acid; Figure S3. 3D diagrams of the effects of process parameters on the formation of narirutin in SWE extracts of mandarin peel (*Citrus unshiu* var. *Kuno*); Figure S4. 3D diagrams of the effects of process parameters on the formation of rutin in SWE extracts of mandarin peel (*Citrus unshiu* var. *Kuno*); Figure S5. 3D diagram of the effects of process parameters on the formation of chlorogenic acid in SWE extracts of mandarin peel (*Citrus unshiu* var. *Kuno*); Table S1. Analysis of variance (ANOVA) of second-order polynomial models for 5-HMF content in the mandarin peels (*Citrus unshiu* var. *Kuno*) obtained by SWE technique; Table S2. Analysis of variance (ANOVA) of second-order polynomial models for hesperidin content in the mandarin peels (*Citrus unshiu* var. *Kuno*) obtained by SWE technique; Table S3. Analysis of variance (ANOVA) of second-order polynomial models for narirutin content in the mandarin peels (*Citrus unshiu* var. *Kuno*) obtained by SWE technique; Table S4. Analysis of variance (ANOVA) of second-order polynomial models for rutin content in the mandarin peels (*Citrus unshiu* var. *Kuno*) obtained by SWE technique; Table S5. Analysis of variance (ANOVA) of reduced third-order polynomial models for chlorogenic acid content in the mandarin peels (*Citrus unshiu* var. *Kuno*) obtained by SWE technique.
