# Supplementary material for: Green Extraction Techniques for Obtaining Bioactive Compounds from Mandarin Peel (Citrus unshiu var. Kuno): Phytochemical Analysis and Process Optimization
Source: Foods. 2021 May 11;10(5):1043. doi: 10.3390/foods10051043 (PMC8150917; doi:10.3390/foods10051043)
Supplement: Supplementary file 1 [file foods-10-01043-s001.zip › foods-1189054-supplementary.pdf]

Supplementary Material for

Green Extraction Techniques for Obtaining Bioactive Compounds from Mandarin Peel (*Citrus unshiu* var. *Kuno*): Phytochemical Analysis and Process Optimization

Silvija Šafranko[1], Ina Ćorković[1], Igor Jerković[2], Martina Jakovljević[1], Krunoslav Aladić[1], Drago Šubarić[1], Stela Jokić[1]*

[1]Faculty of Food Technology Osijek, University of Osijek, Franje Kuhača 18, 31000 Osijek, Croatia; silvija.safranko@ptfos.hr (S.Š.); ina.corkovic@ptfos.hr (I.Ć.); mjakovljevic@ptfos.hr (M.J.); kaladic@ptfos.hr (K.A.) dsubaric@ptfos.hr (D.Š.); sjokic@ptfos.hr (S.J.)

[2]Faculty of Chemistry and Technology, University of Split, Ruđera Boškovića 35, 21000 Split, Croatia; igor@ktf-split.hr (I.J.)

*Correspondence: e-mail: sjokic@ptfos.hr; Tel.: 00385 31 224 333 (S.J.)

Figure captions:

Figure S1. HPLC chromatograms (selected sample 8 sample 17).

Figure S2. Comparison of UV spectra of standards and unknown peaks; A) hesperidin, B) narirutin, C) rutin, D) 5-HMF, and E) chlorogenic acid.

Figure S3. 3D diagrams of the effects of process parameters on the formation of narirutin in SWE extracts of mandarin peel (*Citrus unshiu* var. *Kuno*).

Figure S4. 3D diagrams of the effects of process parameters on the formation of rutin in SWE extracts of mandarin peel (*Citrus unshiu* var. *Kuno*).

Figure S5. 3D diagram of the effects of process parameters on the formation of chlorogenic acid in SWE extracts of mandarin peel (*Citrus unshiu* var. *Kuno*).

Figure S1

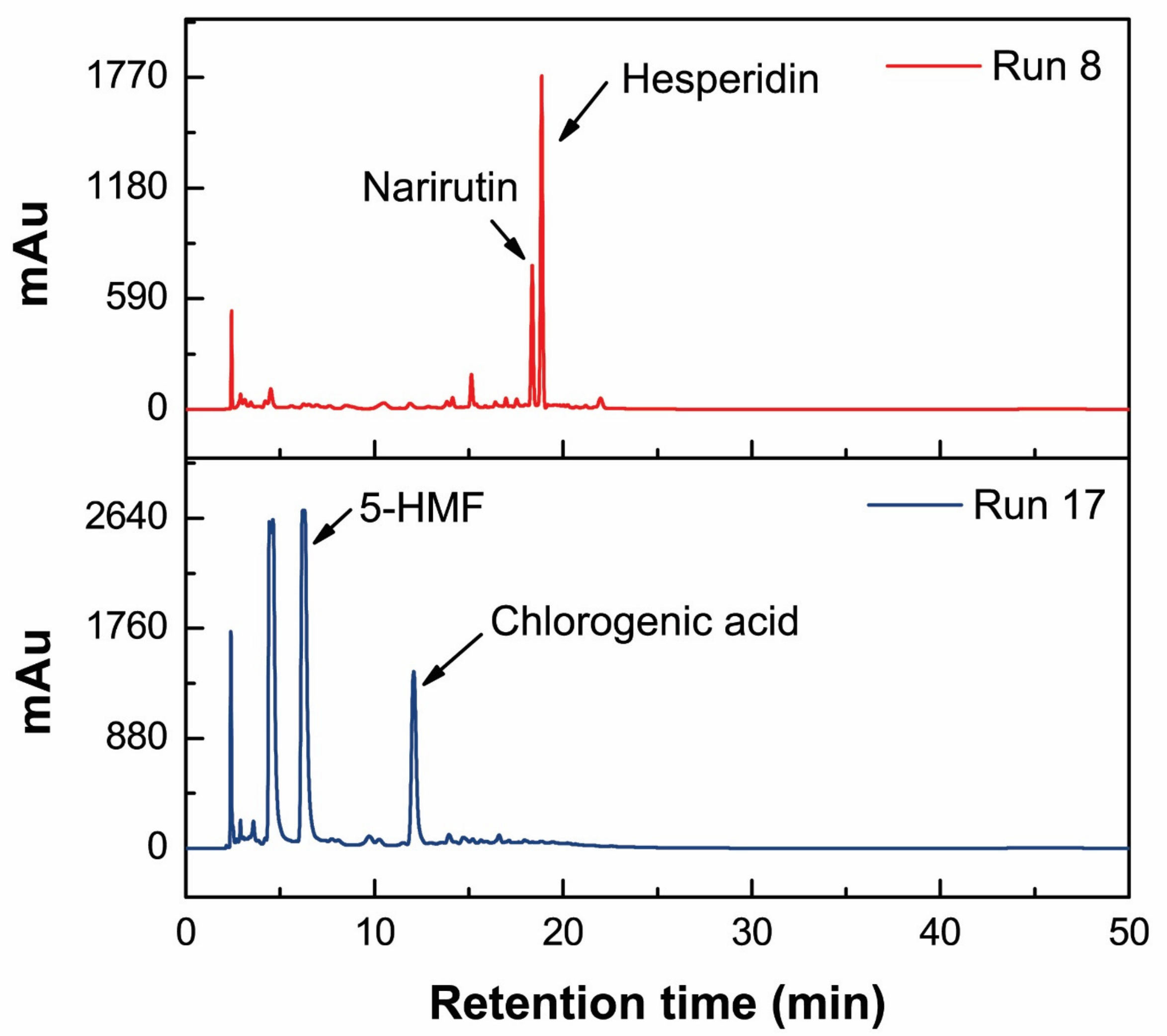

Run 8
Hesperidin
Narirutin
1770
1180
590
0
mAu
Run 17
5-HMF
Chlorogenic acid
2640
1760
880
0
mAu
0
10
20
30
40
50
Retention time (min)

Figure S2

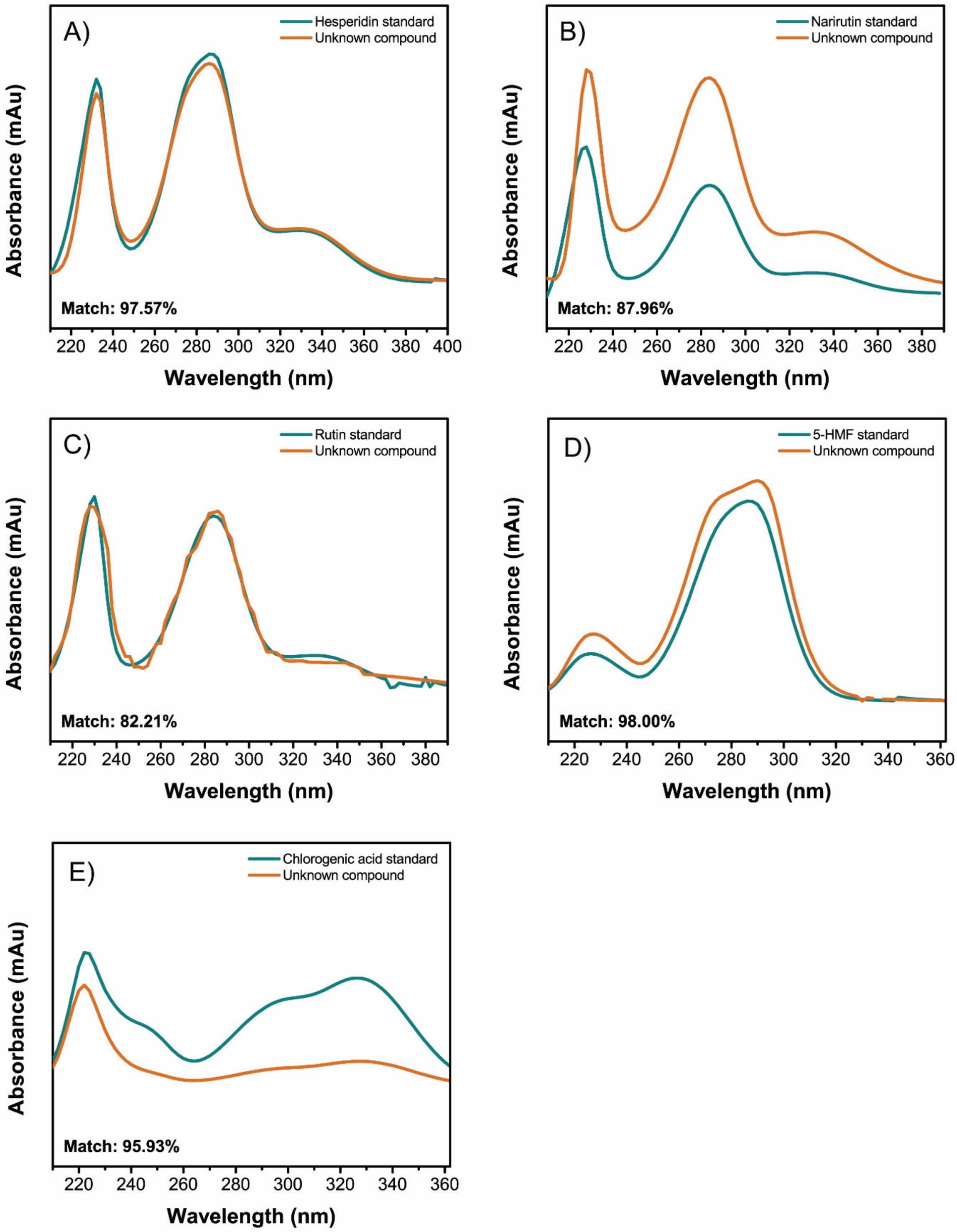
A)
Hesperidin standard
Unknown compound
Match: 97.57%
Absorbance (mAu)
Wavelength (nm)
220 240 260 280 300 320 340 360 380 400
B)
Narirutin standard
Unknown compound
Match: 87.96%
Absorbance (mAu)
Wavelength (nm)
220 240 260 280 300 320 340 360 380
C)
Rutin standard
Unknown compound
Match: 82.21%
Absorbance (mAu)
Wavelength (nm)
220 240 260 280 300 320 340 360 380
D)
5-HMF standard
Unknown compound
Match: 98.00%
Absorbance (mAu)
Wavelength (nm)
220 240 260 280 300 320 340 360
E)
Chlorogenic acid standard
Unknown compound
Match: 95.93%
Absorbance (mAu)
Wavelength (nm)
220 240 260 280 300 320 340 360

Figure S3

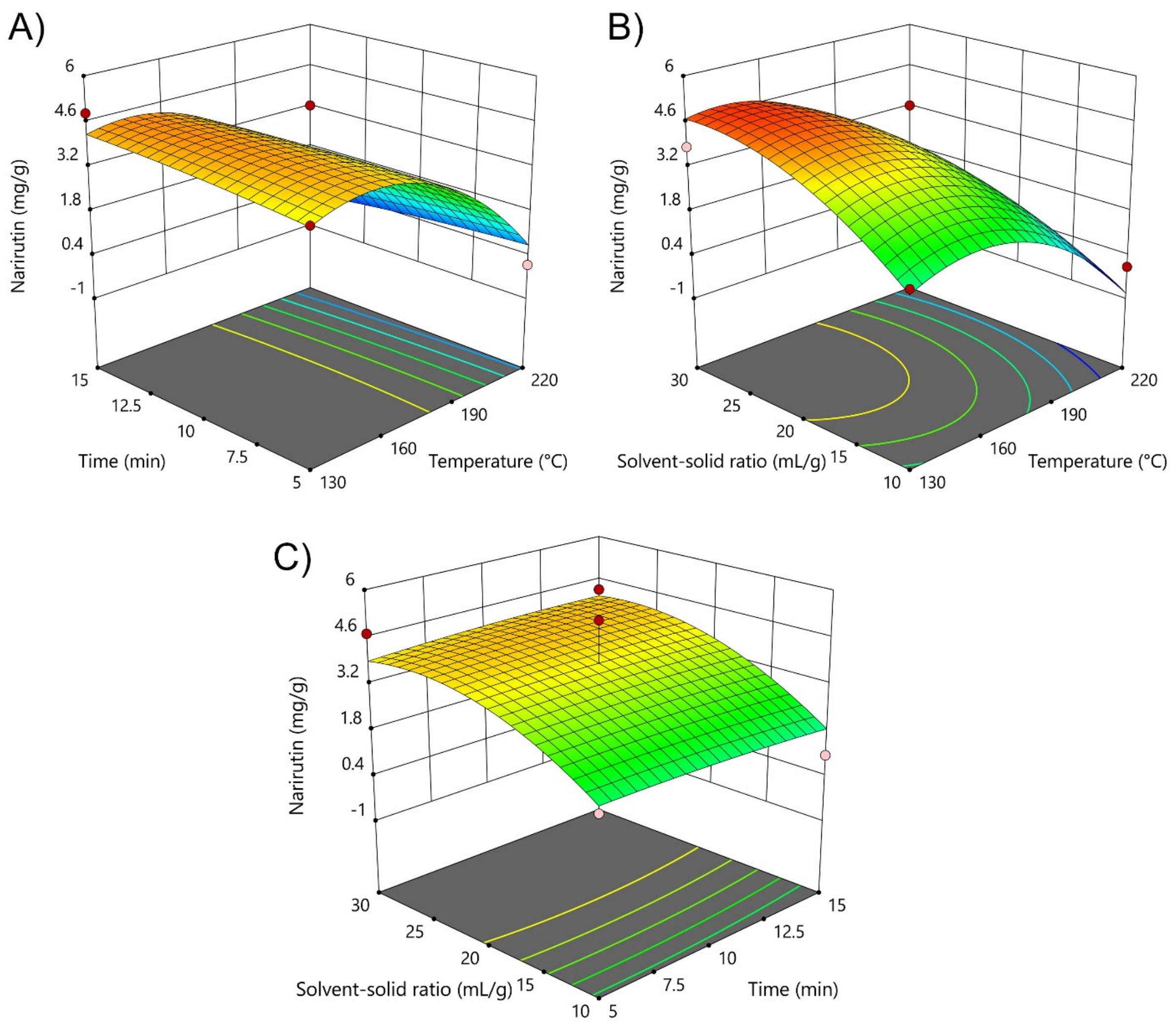

A)
Narirutin (mg/g)
6
4.6
3.2
1.8
0.4
-1
Time (min)
15
12.5
10
7.5
5
Temperature (°C)
130
160
190
220
B)
Narirutin (mg/g)
6
4.6
3.2
1.8
0.4
-1
Solvent-solid ratio (mL/g)
30
25
20
15
10
Temperature (°C)
130
160
190
220
C)
Narirutin (mg/g)
6
4.6
3.2
1.8
0.4
-1
Solvent-solid ratio (mL/g)
30
25
20
15
10
Time (min)
5
7.5
10
12.5
15

Figure S4

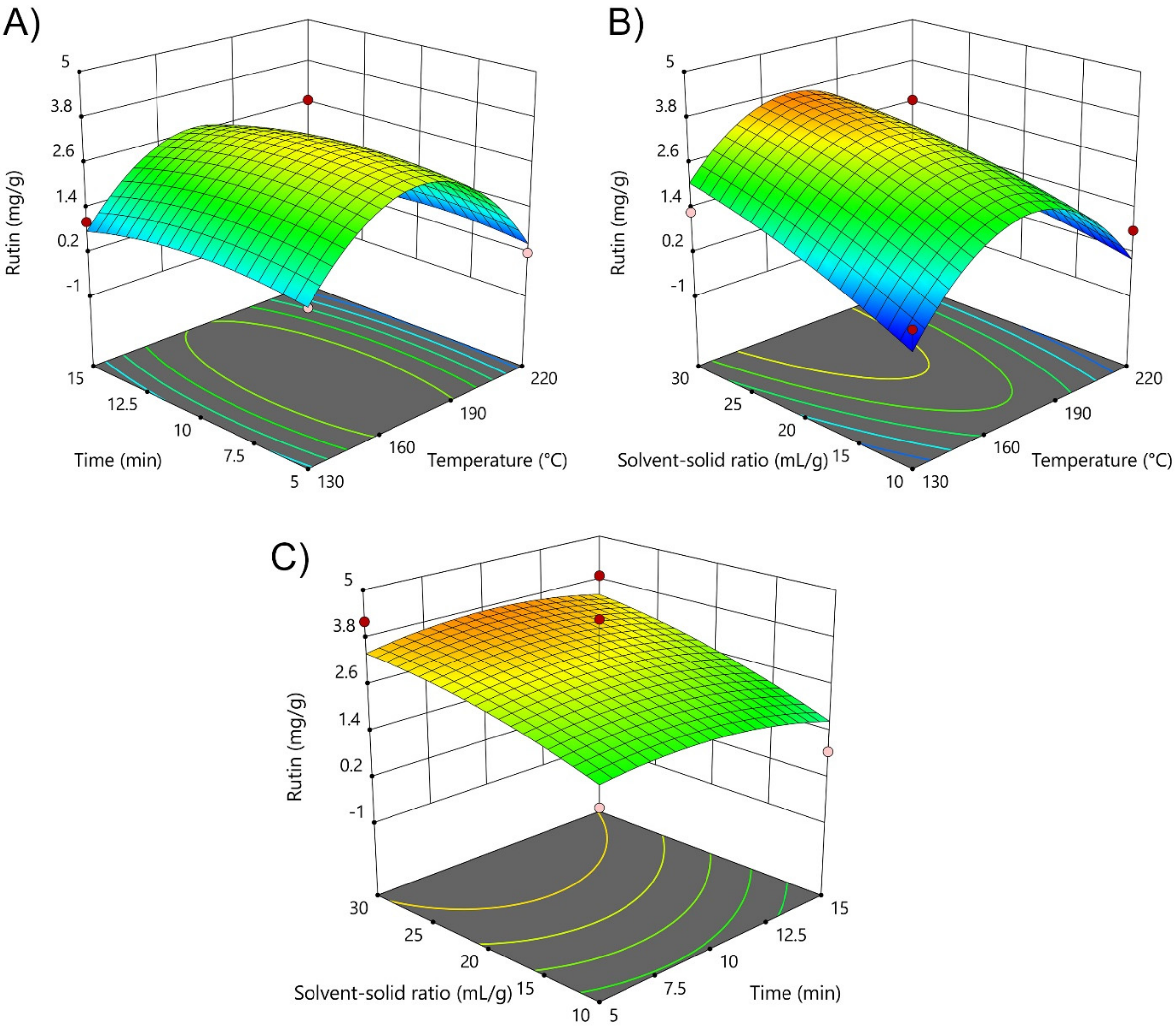

A)
5
3.8
2.6
1.4
0.2
-1
Rutin (mg/g)
15
12.5
10
7.5
5
Time (min)
130
160
190
220
Temperature (°C)
B)
5
3.8
2.6
1.4
0.2
-1
Rutin (mg/g)
30
25
20
15
10
Solvent-solid ratio (mL/g)
130
160
190
220
Temperature (°C)
C)
5
3.8
2.6
1.4
0.2
-1
Rutin (mg/g)
30
25
20
15
10
Solvent-solid ratio (mL/g)
5
7.5
10
12.5
15
Time (min)

Figure S5

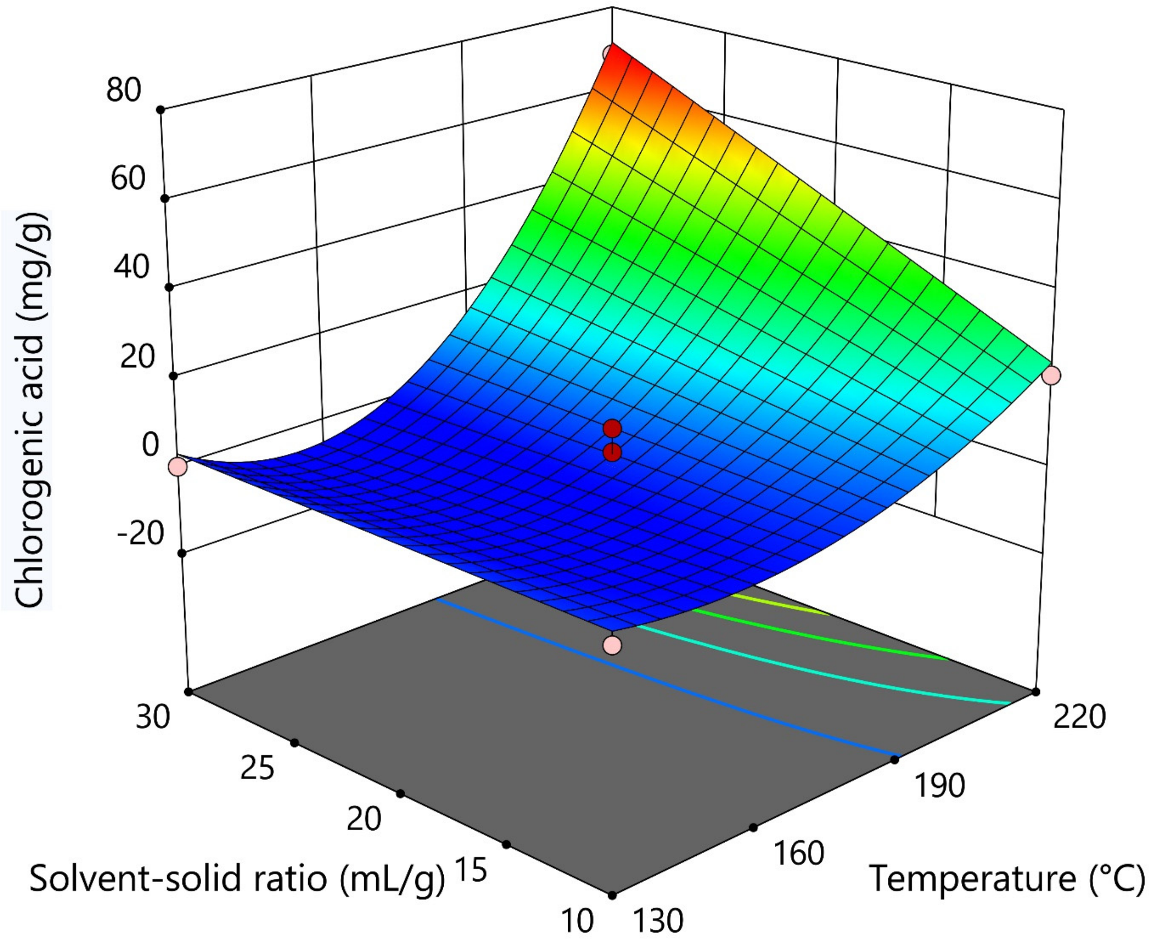
80
60
40
20
0
-20
Chlorogenic acid (mg/g)
30
25
20
15
10
Solvent-solid ratio (mL/g)
130
160
190
220
Temperature (°C)

Table S1. Analysis of variance (ANOVA) of second-order polynomial models for 5-HMF content in the mandarin peels (*Citrus unshiu* var. *Kuno*) obtained by SWE technique.

**Source**	**Sum of Squares**	**Degree of Freedom (df)**	**Mean Square**	**F Value**	***p*-Value**[a]
Model	241.09	9	26.79	23.29	0.0002
X_1 - temperature	163.24	1	163.24	141.91	<0.0001
X_2 - time	0.2552	1	0.2552	0.22	0.6520
X_3 – solvent-solid ratio	41.72	1	41.72	36.27	0.0005
X_1X_2	3.35	1	3.35	2.91	0.1316
X_1X_3	19.95	1	19.95	17.35	0.0042
X_2X_3	0.25	1	0.25	0.22	0.6553
X_1^2	10.93	1	10.93	9.50	0.0177
X_2^2	0.20	1	0.20	0.17	0.6886
X_3^2	1.45	1	1.45	1.26	0.2984
Residual	8.05	7	1.15		
Lack of fit	5.88	3	1.96	3.60	0.1238
Pure error	2.18	4	0.5439		
Total	249.15	16			
R^2	0.9677				

[a] $p < 0.01$ highly significant; $0.01 \leq p < 0.05$ significant; $p \geq 0.05$ not significant.

Table S2. Analysis of variance (ANOVA) of second-order polynomial models for hesperidin content in the mandarin peels (*Citrus unshiu* var. *Kuno*) obtained by SWE technique.

**Source**	**Sum of Squares**	**Degree of Freedom (df)**	**Mean Square**	**F Value**	***p*-Value**[a]
Model	371.66	9	41.30	10.61	0.0026
X_1 - temperature	109.44	1	109.44	28.11	0.0011
X_2 - time	6.10	1	6.10	1.57	0.2510
X_3 – solvent-solid ratio	54.30	1	54.30	13.95	0.0073
X_1X_2	3.87	1	3.87	0.9942	0.3519
X_1X_3	10.05	1	10.05	2.58	0.1522
X_2X_3	2.98	1	2.98	0.7649	0.4108
X_1^2	182.13	1	182.13	46.78	0.0002
X_2^2	2.59	1	2.59	0.6663	0.4412
X_3^2	0.55	1	0.5538	0.1422	0.7172
Residual	27.26	7	3.89		
Lack of fit	22.23	3	7.41	5.90	0.0597
Pure error	5.03	4	1.26		
Total	398.91	16			
R^2	0.9317				

[a] $p < 0.01$ highly significant; $0.01 \le p < 0.05$ significant; $p \ge 0.05$ not significant.

Table S3. Analysis of variance (ANOVA) of second-order polynomial models for narirutin content in the mandarin peels (*Citrus unshiu* var. *Kuno*) obtained by SWE technique.

**Source**	**Sum of Squares**	**Degree of Freedom (df)**	**Mean Square**	**F Value**	***p*-Value**
Model	47.72	9	5.30	6.35	0.0118
X_1 - temperature	24.99	1	24.99	29.90	0.0009
X_2 - time	0.0058	1	0.0058	0.0069	0.9362
X_3 – solvent-solid ratio	7.90	1	7.90	9.45	0.0180
X_1X_2	0.3102	1	0.3102	0.3712	0.5616
X_1X_3	0.7676	1	0.7676	0.9187	0.3696
X_2X_3	0.0436	1	0.0436	0.0522	0.8258
X_1^2	10.36	1	10.36	12.40	0.0097
X_2^2	0.0239	1	0.0239	0.0286	0.8705
X_3^2	2.58	1	2.58	3.09	0.1221
Residual	5.85	7	0.8356		
Lack of fit	3.79	3	1.26	2.46	0.2026
Pure error	2.06	4	0.5142		
Total	53.57	16			
R^2	0.8908				

[a] $p < 0.01$ highly significant; $0.01 \leq p < 0.05$ significant; $p \geq 0.05$ not significant.

Table S4. Analysis of variance (ANOVA) of second-order polynomial models for rutin content in the mandarin peels (*Citrus unshiu* var. *Kuno*) obtained by SWE technique.

**Source**	**Sum of Squares**	**Degree of Freedom (df)**	**Mean Square**	**F Value**	***p*-Value**[a]
Model	29.11	9	3.23	4.36	0.0325
X_1 - temperature	0.7642	1	0.7642	1.03	0.3437
X_2 - time	0.1556	1	0.1556	0.21	0.6607
X_3 – solvent-solid ratio	4.20	1	4.20	5.67	0.0489
X_1X_2	0.00	1	0.00	0.00	0.9964
X_1X_3	0.4424	1	0.4424	0.5969	0.4650
X_2X_3	0.0605	1	0.0605	0.0817	0.7833
X_1^2	22.01	1	22.01	29.70	0.0010
X_2^2	0.4767	1	0.4767	0.6432	0.4489
X_3^2	0.2183	1	0.2183	0.2946	0.6042
Residual	5.19	7	0.7411		
Lack of fit	3.79	3	1.26	3.61	0.1233
Pure error	1.40	4	0.3496		
Total	34.29	16			
R^2	0.8487				

[a] $p < 0.01$ highly significant; $0.01 \leq p < 0.05$ significant; $p \geq 0.05$ not significant.

Table S5. Analysis of variance (ANOVA) of reduced third-order polynomial models for chlorogenic acid content in the mandarin peels (*Citrus unshiu* var. *Kuno*) obtained by SWE technique.

**Source**	**Sum of Squares**	**Degree of Freedom (df)**	**Mean Square**	**F Value**	***p*-Value**[a]
Model	8469.29	8	1058.66	63.19	< 0.0001
X_1-temperature	2001.42	1	2001.42	119.47	< 0.0001
X_1X_3	555.10	1	555.10	33.13	0.0004
X_1^2	2111.05	1	2111.05	126.01	< 0.0001
$X_1^2X_3$	564.58	1	564.58	33.70	0.0004
$X_1X_2^2$	69.88	1	69.88	4.17	0.0754
$X_2^2X_3$	9.87	1	9.87	0.5893	0.4648
$X_2X_3^2$	25.46	1	25.46	1.52	0.2527
X_2^3	5.64	1	5.64	0.3364	0.5779
Residual	134.02	8	16.75		
Lack of fit	87.19	4	21.80	1.86	0.2810
Pure error	46.83	4	11.71		
Total	8603.31	16			
R^2	0.9844				

[a] $p < 0.01$ highly significant; $0.01 \leq p < 0.05$ significant; $p \geq 0.05$ not significant.
